# A brain somatic *RHEB* doublet mutation causes focal cortical dysplasia type II

**DOI:** 10.1038/s12276-019-0277-4

**Published:** 2019-07-23

**Authors:** Shanshan Zhao, Zhenghui Li, Muxian Zhang, Lingliang Zhang, Honghua Zheng, Jinhuan Ning, Yanyan Wang, Fengpeng Wang, Xiaobin Zhang, Hexia Gan, Yuanqing Wang, Xian Zhang, Hong Luo, Guojun Bu, Huaxi Xu, Yi Yao, Yun-wu Zhang

**Affiliations:** 1 Fujian Provincial Key Laboratory of Neurodegenerative Disease and Aging Research, Institute of Neuroscience, School of Medicine, Xiamen University, Xiamen, 361102 Fujian China; 2 Neuromedicine Center, the 174th Hospital of Chinese People’s Liberation Army, Affiliated Chenggong Hospital, Xiamen University, Xiamen, 361003 Fujian China; 3 Department of Neurosurgery, Kaifeng Central Hospital, Kaifeng, 475000 Henan China; 4 XiaMen Humanity Hospital, No.3777 XianYue Road, HuLi District, XiaMen, 361015 FuJian China; 5 Department of Neuroscience, Mayo Clinic, Jacksonville, FL 32224 USA; 6 Neuroscience Initiative, Sanford-Burnham-Prebys Medical Discovery Institute, La Jolla, CA 92037 USA; 7 Department of Pediatric Neurology, Shenzhen Children’s Hospital, Shenzhen, 518026 Guangdong Province China

**Keywords:** Epilepsy, Disease genetics, Genetics of the nervous system

## Abstract

Focal cortical dysplasia type II (FCDII) is a cerebral cortex malformation characterized by local cortical structure disorganization, neuronal dysmorphology, and refractory epilepsy. Brain somatic mutations in several genes involved in the PI3K/AKT/mTOR pathway are associated with FCDII, but they are only found in a proportion of patients with FCDII. The genetic causes underlying the development FCDII in other patients remain unclear. Here, we carried out whole exome sequencing and targeted sequencing in paired brain–blood DNA from patients with FCDII and identified a brain somatic doublet mutation c.(A104T, C105A) in the Ras homolog, mTORC1 binding (*RHEB*) gene, which led to the RHEB p.Y35L mutation in one patient with FCDII. This *RHEB* mutation carrier had a dramatic increase of ribosomal protein S6 phosphorylation, indicating mTOR activation in the region of the brain lesion. The RHEB p.Y35L mutant protein had increased GTPλS-binding activity compared with wild-type RHEB. Overexpression of the RHEB p.Y35L variant in cultured cells also resulted in elevated S6 phosphorylation compared to wild-type RHEB. Importantly, in utero electroporation of the RHEB p.Y35L variant in mice induced S6 phosphorylation, cytomegalic neurons, dysregulated neuron migration, abnormal electroencephalogram, and seizures, all of which are found in patients with FCDII. Rapamycin treatment rescued abnormal electroencephalograms and alleviated seizures in these mice. These results demonstrate that brain somatic mutations in *RHEB* are also responsible for the pathogenesis of FCDII, indicating that aberrant activation of mTOR signaling is a primary driver and potential drug target for FCDII.

## Brain development disorder: causal mutation suggests target for treating epilepsy

Identifying a genetic mutation causing a congenital brain disorder that triggers difficult-to-treat epilepsy suggests a potential therapeutic target, according to Chinese scientists. Focal cortical dysplasia type II (FCDII) is one of a group of brain development abnormalities that cause intractable epilepsy. Scientists have identified gene mutations in some FCDII patients linked to a signaling pathway involved in cell proliferation and metabolism in the developing brain. Now, Yun-wu Zhang and Yi Yao at Xiamen University in Fujian, China, and co-workers have discovered a mutation on a gene which triggers abnormal activation of the same signaling pathway. The team found that the drug rapamycin, which inhibits this pathway, alleviated epileptic seizures in mice with the mutant gene. Their findings add weight to the theory that this pathway may be a viable target for future therapies.

## Introduction

Focal cortical dysplasia (FCD) is a cortical developmental malformation associated with intractable epilepsy in children. FCD is classified into three types: FCDI refers to cortical structural abnormalities in the absence of cell abnormalities. FCDII is characterized by disrupted cortical lamination and aberrant neuronal morphology (such as cytomegalic neurons in both FCDIIa and FCDIIb and balloon cells exclusively in FCDIIb). FCDIII involves abnormalities in cortical structures associated with lesions^1–3^. Although surgical resection of the epileptic focus may correct seizure onset in a large proportion of patients with FCD, seizures still persist in other patients with FCD^4^. Hence, determining the molecular mechanisms underlying FCD is fundamental to developing complementary treatment strategies.

The PI3K/AKT/mTOR signaling pathway plays important roles in cell proliferation, migration, and metabolism during brain development^5,6^. Recent studies have identified brain somatic mutations in several genes in this pathway in association with FCDII, including *PIK3CA*^7^, *PTEN*^8^, *DEPDC5*^9^, *TSC1*^10,11^, *TSC2*^10,11^, and *MTOR*^11–15^. However, somatic mutations in these genes are only found in a proportion of patients with FCDII. For example, somatic mutations in *MTOR* were only identified in 7.7^11^, 46.2^12^, 15.6^13^, 13.8^14^, and 37.5%^15^ of participants in different studies. Somatic mutations in *TSC1* were identified in 10.0% of participants in one study^10^. Somatic mutations in other genes are even scarcer, with only one case identified in each study. Therefore, the genetic causes underlying the development of FCDII in other mTOR-related and, possibly, mTOR-independent genes have yet to be determined. Here, we describe a new pathogenic mutation in the Ras homolog, mTORC1-binding (*RHEB*) gene associated with mTOR pathway activation, providing evidence of an additional somatic mTOR modulator in FCDII.

## Materials and methods

### Human subjects

Eight patients with FCDII were subjected to a preoperative clinical evaluation, including magnetic resonance imaging (MRI), positron emission tomography (PET) scanning, seizure chart review, and electroencephalography (EEG). After surgical treatment, fresh resected specimens were stored at −80 °C. A portion of the samples were subjected to hematoxylin-eosin (H&E) staining to confirm the existence of the dysmorphic neurons characteristically observed in FCDII. Patient information is listed in Table 1. Patient blood samples were collected immediately prior to DNA extraction. Control brain tissue samples were derived from patients with non-FCD diseases (craniocerebral injury, arterial aneurysm, or intracerebral hemorrhage) and were pathologically negative for FCD. Study protocols received prior approval from the Ethics Committee of the 174th Hospital of Chinese People’s Liberation Army, Affiliated Chenggong Hospital, Xiamen University and Medical College of Xiamen University. Informed content was obtained from all subjects.

**Table 1:** Patient information

Patient	Age at surgery	Sex	Pathology	MRI report
FCDII-1	4 years, 4 months	M	Gray matter not clear; dysmorphic neurons, consistent with FCDII	Cortical dysplasia involving the right mesial premotor gyrus
FCDII-2	2 years, 4 months	M	Cortical dyslamination, dysmorphic neurons, and balloon cells, consistent with FCDIIb	Cortical dysplasia involving the right premotor, precentral, and central gyrus
FCDII-3	5 years, 3 months	F	Cortical dyslamination and dysmorphic neurons, consistent with FCDII	Cortical dysplasia involving the right mesial premotor gyrus
FCDII-4	11 years, 11 months	F	Cortical dyslamination and dysmorphic neurons, consistent with FCDII	Cortical dysplasia involving the right postcentral gyrus
FCDII-5	2 years, 7 months	F	Gray matter not clear; dysmorphic neurons, consistent with FCDII	Cortical dysplasia involving the left premotor, central gyrus
FCDII-6	16 years, 4 months	M	Cortical dyslamination, dysmorphic neurons, and balloon cells, consistent with FCDIIb	Cortical dysplasia involving the left occipital gyrus
FCDII-7	1 years, 1 months	M	Cortical dyslamination and dysmorphic neurons, consistent with FCDII	Cortical dysplasia involving the right insular and frontal operculum gyrus
FCDII-8	2 years, 2 months	M	Cortical dyslamination and dysmorphic neurons, consistent with FCDII	Cortical dysplasia involving the right insular and parietal operculum gyrus

### H&E staining and immunohistochemistry

Surgical tissues were fixed overnight in 4% paraformaldehyde and sequentially cryoprotected in 20, 25, and 30% sucrose buffer; embedded in OCT compound; and stored at −80 °C. Cryostat sections (15 μm thick) were collected and placed on glass slides. Sections were stained with H&E. Alternatively, sections were first treated with pH 6.0 citric acid (Fuzhou Maixin Biotech, Fuzhou, Fujian, China) for 10 min at high temperature for antigen retrieval, permeabilized in 0.2% Triton X-100, and subjected to immunohistochemistry using an antibody against the S6 ribosomal protein phosphorylated at Ser240/Ser244 (5364S, Cell Signaling Technology, Danvers, MA, USA) in combination with the UltraSensitive^TM^ SP IHC Kit (Fuzhou Maixin Biotech) following the manufacturer’s protocol.

### Whole exome sequencing (WES) and targeted sequencing

Genomic DNA was extracted from brain and blood samples using the DNeasy Blood & Tissue Kit (Qiagen, Shanghai, China). For WES, genomic DNA from four patients with FCDII (FCDII-1 to FCDII-4) was subjected to whole exome capture library construction using the SureSelect Human ALL Exon V5 Kit (Agilent, Beijing, China). Libraries were subjected to paired-end sequencing using the Illumina HiSeq platform (Illumina, San Diego, CA, USA).

For targeted sequencing, genomic DNA from eight patients with FCDII (FCDII-1 to FCDII-8) were fragmented into small pieces and subjected to barcoded library construction. Targeted region capture was performed using the designed target region bed including *MTOR*, *RHEB*, *TSC1*, *TSC2*, *PTEN*, *DEPDC5*, and *PIK3CA*. Paired-end sequencing was performed using the Illumina HiSeq X-ten instruments.

### Data processing and bioinformatic analysis

Raw sequencing reads were filtered by removing adapter reads, reads with an N (undetermined nucleotide) ratio higher than 10%, and paired reads when the number of low quality (<5) nucleotides in one of the paired reads was over 50% of the read length. Clean reads were mapped to the human genome reference (GRCh37/hg19) using the Burrows–Wheeler Aligner^16^. After sorting with SAMtools^17^, data files were processed to mark duplicates using Picard (http://sourceforge.net/projects/picard/). Paired data (brain and blood) were analyzed using MuTect^18^, Strelka^19^ and control-FREEC^20^ to detect de novo somatic single nucleotide variants (SNVs), indels and copy number variations (CNVs) in brain tissues, respectively. Called variants were annotated using ANNOVAR^21^. The identified somatic mutations were given a high priority and subjected to further functional analyses when they were located within exonic or splicing regions but not genome repeat regions, had low frequencies (<0.01) in both the 1000 Genomes Project Consortium Database^22^ and the Genome Aggregation Database (gnomAD)^23^, and were predicted to be pathogenic by SIFT^24^, Polyphen^25^, MutationTaster^26^, and/or CADD^27^.

### *RHEB* mutation validation

In addition to the portion used for WES, genomic DNA was extracted from another portion of the *RHEB* mutation-carrier lesion region and used as a PCR template to amplify an *RHEB* region spanning the mutation site using forward (5′-TGTTTTCTGACCCTGATACCTT-3′) and reverse (5′-TGCAAGTTTTCTTTGACCCCA-3′) primer pairs. PCR products were cloned into the pMD19 T-vector (Takara Bio). Individual T-vector clones were subjected to Sanger sequencing.

### Plasmids, transfection, and western blotting

Wild-type and c.(A104T, C105A) double mutant human *RHEB* cDNA were cloned into the pcAGIG vector containing the IRES-GFP reporter. The plasmids were transfected into HEK293T cells using the Turbofect reagent (Thermo Fisher Scientific, Shanghai, China). Transfected cells and resected FCDII and non-FCD control patient tissues were lysed in RIPA buffer. Protein lysates were analyzed by western blotting with various antibodies, including anti-phosphorylated S6 (5364S, Cell Signaling Technology), anti-total S6 (2217S, Cell Signaling Technology), anti-RHEB (15924-1-AP, Proteintech Group, Rosemont, IL, USA), anti-phosphorylated mTOR (CY6571, Ser2448, Abways Technology, Shanghai, China), anti-total mTOR (CY5306, Abways Technology), and anti-α-tubulin (MABT205, Millipore, Beijing, China). The protein band intensity was quantified using ImageJ.

### RHEB-GTPγS binding assay

Recombinant WT and mutant RHEB proteins were generated by Sino Biological (Beijing, China). The GTP analog BODIPY-GTPγS (G22183) was from Thermo Fisher Scientific. To detect their binding, 1-μM recombinant RHEB protein was added to reaction buffer [50 mM Tris-HCl (pH 8.0), 1 mM EDTA, and 10 mM MgCl_2_] and incubated at room temperature for 15 min, and then, 50 nM BODIPY-GTPγS was added. The intensity of the fluorescence emission was recorded every 10 s at 30 °C with a Varioskan Flash plate reader (Thermo Fisher Scientific) under excitation at 485 nm and emission at 528 nm with a bandwidth of 5 nm. The relative fluorescence was calculated as:

$$\begin{array}{l}{\mathrm{RF}} = \frac{{\Delta F}}{{F0}} = \\ \frac{\begin{array}{l}\left( {{\mathrm{fluorescence}}\,{\mathrm{from}}\,{\mathrm{BODIPYGTP\gamma SRHEB}}} \right)\\ -\, \left( {{\mathrm{fluorescence}}\,{\mathrm{from}}\,{\mathrm{BODIPYGTP\gamma S}}\,{\mathrm{alone}}} \right)\end{array}}{{{\mathrm{fluorescence}}\,{\mathrm{from}}\,{\mathrm{BODIPYGTP\gamma S}}\,{\mathrm{alone}}}}\end{array}$$

### In utero electroporation and image analysis

Pregnant ICR mice at embryonic day 14.5 (E14.5) were anesthetized. The uterine horns were exposed, and Fast Green mixed with the vehicle, wild-type or mutant *RHEB* plasmids coexpressing GFP were injected into the embryo ventricle using pulled glass capillaries. The plasmids were electroporated into the embryo head. Mouse brains were harvested at E18.5 or at approximately postnatal day 30 (P30). Cryostat brain sections (15 µm thick) were immunostained with the anti-phosphorylated S6 antibody described above or an anti-NeuN antibody (ab177487, Abcam, Shanghai, China) and a fluorescence-conjugated secondary antibody (A11012, Thermo Fisher Scientific). Images were obtained using a confocal microscope (FV10MPE-B, Olympus, Shanghai, China; or LSM880+Airyscan, Zeiss, Shanghai, China). GFP-positive cells in different cortical layers, with different cell soma sizes and different colocalization between GFP and phosphorylated S6 or NeuN were analyzed using ImageJ. The animal experimentation protocols were approved by the institutional Animal Care and Use Committee of Xiamen University. All studies were conducted in accordance with the United States Public Health Service’s Policy on Humane Care and Use of Laboratory Animals.

### Rapamycin treatment, EEG recording, and behavior observation

Some mouse embryos subjected to in utero electroporation were allowed to be delivered. Among them, some at P30 were closely observed for seizure behaviors and then subjected to EEG recording, and some at 1.5 months old were first treated with rapamycin (HY-10219, MedChemExpress, Shanghai, China) at 10 mg/kg for 11 days and then subjected to the seizure study and EEG recording. EEG data were analyzed using Easy EEG II (version 2.0.1).

### *MED16* siRNA transfection

HEK293T cells were transfected with a scrambled control siRNA (sense 5′-UUCUCCGAACGUGUCACGUTT-3′) or various *MED16* siRNAs (MED16-homo-1: sense 5′-GGUCCUGCCGAAAUCUCAUTT-3′; MED16-homo-2: sense 5′-GCUGCACAAUGGUGUGAAATT-3′; and MED16-homo-3: sense 5′-GCGACAAACAGCCCACAAUTT-3′) for 48 h. Cell lysates were analyzed by quantitative real-time PCR and western blotting.

### Quantitative real-time PCR (qRT-PCR)

Total RNA was isolated using TRIzol reagent (Thermo Fisher Scientific) and transcribed into cDNA using the Rever Tra Ace qPCR RT Kit (TOYOBO, Shanghai, China). qRT-PCR was performed using the FastStart Universal SYBR Green Master (ROX) (Roche, Basel, Switzerland) with specific primers designed for the designated target genes. β-actin was used as an internal control. The primers used for qRT-PCR amplification included human MED16: forward 5′- TGCCGAAATCTCATCGCCTT-3′ and reverse 5′-GCTATTAGCCAGGTGGTCCG-3′; and β-actin: 5′-ATCAAGATCATTGCTCCTCCTGAG-3′ and reverse 5′-CTGCTTGCTGATCCACATCTG-3′.

### Statistical analysis

One-way analysis of variance (ANOVA) with Tukey’s post test, two-way repeated-measures ANOVA or Student’s *t*-test was used for statistical comparisons, where *P* < 0.05 was considered to be statistically significant. Data are presented as the mean ± s.e.m.

## Results

### Somatic mutation screening and validation

**Fig. 1:**
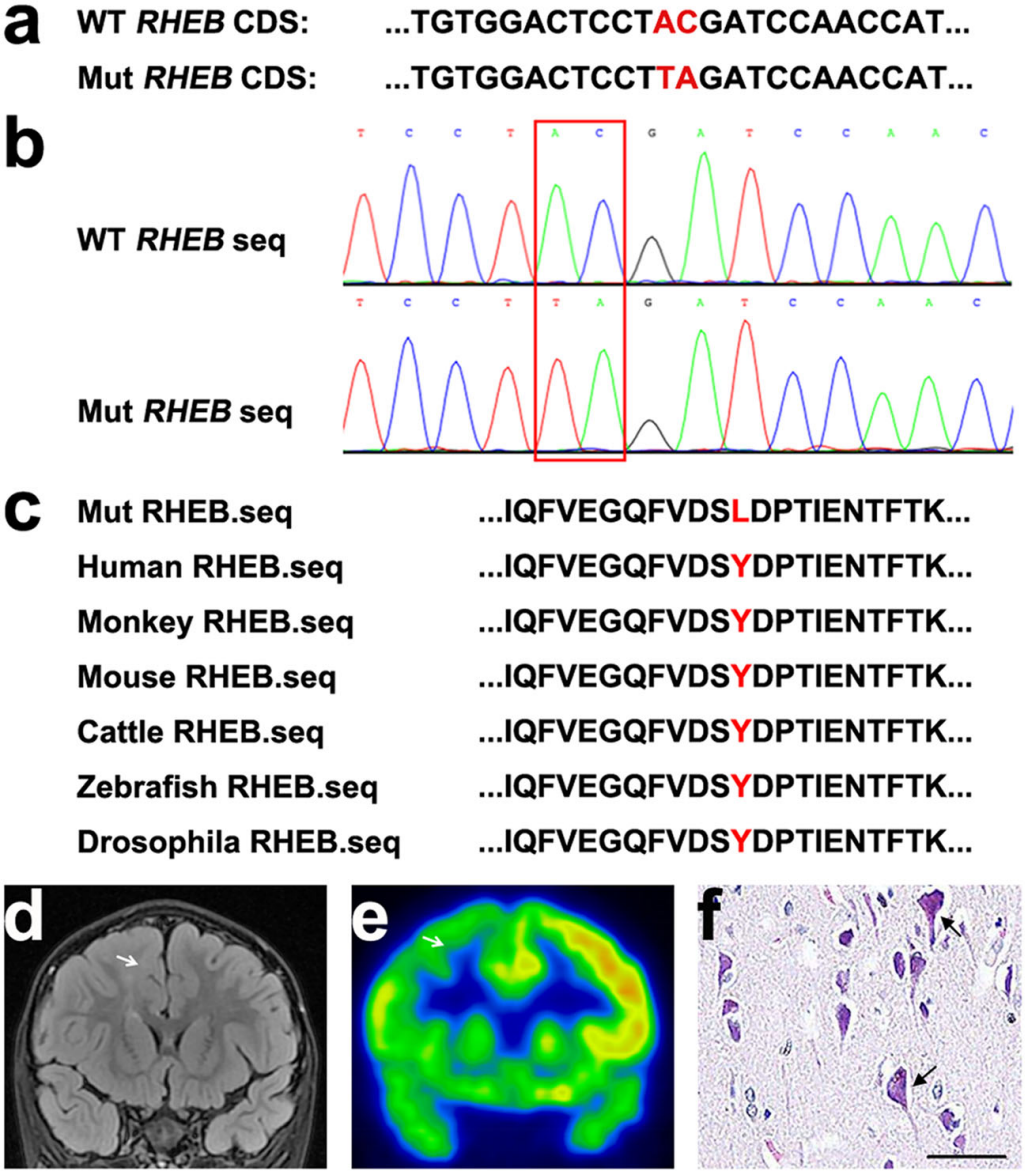
Identification of a brain somatic *RHEB* doublet mutation in a patient with FCDII. **a** Two adjacent *RHEB* mutations (Mut) identified by WES were compared to wild-type (WT) *RHEB*. The mutation sites are highlighted in red. **b** PCR products amplified from DNA derived from patient lesion tissue were subjected to TA cloning. Individual T-vector clones were subjected to Sanger sequencing. The sequencing results of the WT and Mut *RHEB* clones were compared, and the mutation sites are highlighted in red boxes. **c** The RHEB protein regions from different species spanning the mutation site were aligned for comparison. The mutation site is highlighted in red. Brain magnetic resonance imaging (**d**) and positron emission tomography scan (**e**) of the patient carrying the *RHEB* doublet mutation. The white arrows indicate lesion regions. **f** Hematoxylin-eosin staining of resected lesion tissue of the *RHEB* doublet mutation carrier. Abnormal cells are indicated with black arrows. Scale bar: 50 μm

We first carried out WES using paired blood–brain DNA samples from four patients with FCDII (Table 1, FCDII-1 to FCDII-4). The average sequencing depth ranged from 603.56× to 802.85× (Supplementary Table 1). There were 276–371 (median 307) SNVs, 2–12 (median 7.5) indels, and 16–115 (median 52) CNVs identified as brain somatic mutations in the four subjects (Supplementary Table 2). We focused on brain somatic SNVs and indels that fulfilled the high-priority screening criteria, which identified two adjacent SNV mutations (C105A and A104T) in the *RHEB* gene in patient FCDII-1 (Fig. 1a and Table 2) and one SNV in *DPP7* and one indel in *MED16* in patient with FCDII-2 (Table 2).

**Table 2:** Identified brain somatic mutations with high priorities

Sample	Gene name	Chrom: position	Mutation	Mutation type	Amino acid change	Mutation detection frequency comparison (brain vs. blood)
FCDII-1	*RHEB* *RHEB* *RHEB* ^d^	7: 151188048 7: 151188049 7: 151188048,151188049	G→T T→A GT→TA	Stopgain SNV Missense SNV Missense SNVs	RHEB:NM_005614:exon2:c.C105A:p.Y35X RHEB:NM_005614:exon2:c.A104T:p.Y35F RHEB:NM_005614:exon2:c.A104T,C105A:p.Y35L	6.0^a^–6.4%^b^ 5.8^a^–6.3%^b^ NA
FCDII-2	*DPP7* *MED16*	9: 140008691 19: 875274	C→A TCAGCC→T	Splicing SNV Frameshift indel	DPP7:NM_013379:exon4:c.321+4G>T^c^ MED16:NM_005481:exon10:c.1736_1740del:p.579_580del	0.051 (4 in 79) vs. 0 (0 in 52) 0.022 (10 in 447) vs. 0.005 (1 in 217)
FCDII-5	*TSC1*	9: 135782187	T→G	Missense SNV	TSC1:NM_001162427:exon13:c.A1216C:p.S406R; TSC1:NM_000368:exon14:c.A1369C:p.S457R; TSC1:NM_001162426:exon14:c.A1366C:p.S456R	50.1%^a^

^a^Based on the targeted sequencing results

^b^Based on the WES results

^c^The exact amino acid change is not known

^d^Confirmed *RHEB* doublet mutation

The *RHEB* gene encodes Ras homolog enriched in brain (RHEB), a GTP-binding protein involved in cell cycle regulation and an important activator of the mTOR pathway^6,28^. We also carried out targeted sequencing of *RHEB* and other genes, including *MTOR*, *TSC1*, *TSC2*, *PTEN*, *DEPDC5*, and *PIK3CA*, whose somatic mutations were found to cause FCDII, in all eight patients with FCDII to confirm and identify additional FCDII-associated mutations. A mean sequencing depth of 209× to 795× was achieved for these genes. Using the same filtering criteria used for the WES data, we confirmed the existence of the two *RHEB* SNV mutations in patient with FCDII-1 and identified one SNV mutation in *TSC1* (NM_001162427:exon13:c.A1216C:p.S406R; NM_000368:exon14:c.A1369C:p.S457R; NM_001162426:exon14:c.A1366C:p.S456R) in patient with FCDII-5 (Table 2).

Although the initial bioinformatic analysis identified *RHEB* C105A and A104T as two separate mutations (Table 2), integrative genomics viewer analysis of the WES data revealed that the two mutations were always found in the same reads (Supplementary Fig. 1). Moreover, PCR amplified an *RHEB* gene fragment spanning the C105A/A104T mutation sites from this *RHEB* mutation-carrier’s genomic DNA, cloned the PCR products into a TA cloning vector, and randomly sequenced 40 resultant T-vector clones. Two clones were found to include both the C105A/A104T mutations (Fig. 1b), whereas no clone contained a single C105A or A104T mutation, demonstrating that the two *RHEB* mutations occur in *cis* (i.e., on the same allele). The doublet mutation c.(A104T, C105A) results in the RHEB p.Y35L mutation (Fig. 1c).

### Pathological features of the RHEB p.Y35L-carrier

Brain MRI revealed a developmental malformation in the right mesial premotor gyrus of this RHEB p.Y35L-carrying FCDII patient (Fig. 1d). PET analysis showed decreased metabolism within the lesion area (Fig. 1e). H&E staining identified dysmorphic neurons in the surgically resected specimen (Fig. 1f), confirming the diagnosis of FCDII.

### The RHEB p.Y35L mutation leads to aberrant activation of the mTOR pathway

**Fig. 2:**
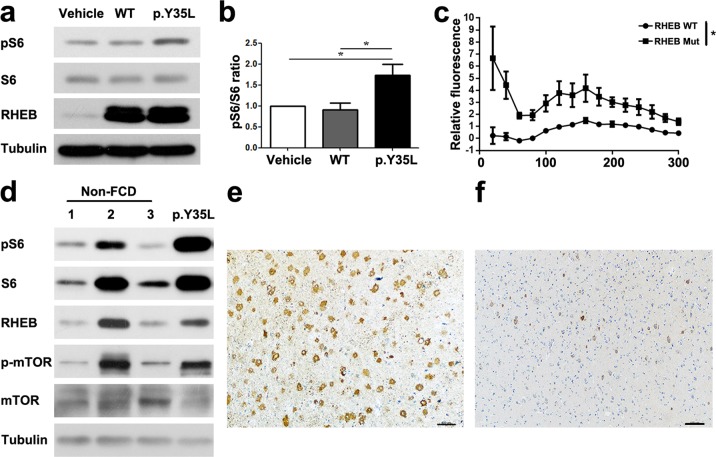
RHEB p.Y35L mutation enhances mTOR activation. **a** Control vehicle, wild-type (WT) and p.Y35L mutant RHEB plasmids were transfected into HEK293T cells. Equivalent protein quantities from cell lysates were subjected to immunoblotting to detect phosphorylated S6 (pS6), total S6, RHEB, and α-tubulin. **b** The protein levels of pS6 were quantified by densitometry and normalized to those of total S6 for comparison, where the pS6/total S6 ratios of the vehicle samples were set as one arbitrary unit. Data are presented as the mean ± s.e.m, *n* = 4, *P < 0.05 (one-way ANOVA with Tukey’s post test). **c** One micromolar recombinant WT or p.Y35L mutant RHEB proteins were mixed with 50 nM BODIPY-GTPγS in reaction buffer. The fluorescence intensity was recorded every 10 s at 30 °C (λ_em_ 485 nm, λ_ex_ 528 nm). Relative fluorescence was calculated for comparison. Data are presented as the mean ± s.e.m, *n* = 3, ^*^P < 0.05 (two-way repeated-measures ANOVA). **d** Brain lysates from three non-FCD controls (1: craniocerebral injury, 2: arterial aneurysm, and 3: intracerebral hemorrhage) and FCDII RHEB p.Y35L mutation carriers were subjected to immunoblotting to detect pS6, total S6, RHEB, phosphorylated mTOR (p-mTOR), total mTOR, and α-tubulin. Immunohistochemical analysis of the brain lesion region of the FCDII RHEB p.Y35L mutation carrier (**e**) and a corresponding brain region from a non-FCD control (**f**). Scale bars: 100 μm

RHEB p.Y35 is highly conserved in diverse species (Fig. 1c). A previous study suggests that RHEB p.Y35 may play an important role in regulating GTP binding^29^. We observed that S6 phosphorylation (a marker of mTOR activation) was significantly increased in cells expressing the RHEB p.Y35L mutant compared with those expressing wild-type RHEB or vehicle (Fig. 2a, b), whereas downregulation of the overexpressed RHEB p.Y35L mutant reduced S6 phosphorylation (Supplementary Fig. 2). In addition, we found that the recombinant RHEB p.Y35L mutant protein had increased GTPγS binding activity compared to wild-type RHEB (Fig. 2c). Moreover, both the level of phosphorylated S6 protein (Fig. 2d) and immunoreactivity (Fig. 2e, f) were markedly increased in the region of the lesion in this *RHEB* mutation carrier compared to non-FCD controls. These results indicate that the RHEB p.Y35L mutation may enhance its activity to promote the mTOR pathway.

### Embryonic expression of the RHEB p.Y35L variant induces FCDII-like phenotypes in mice

**Fig. 3:**
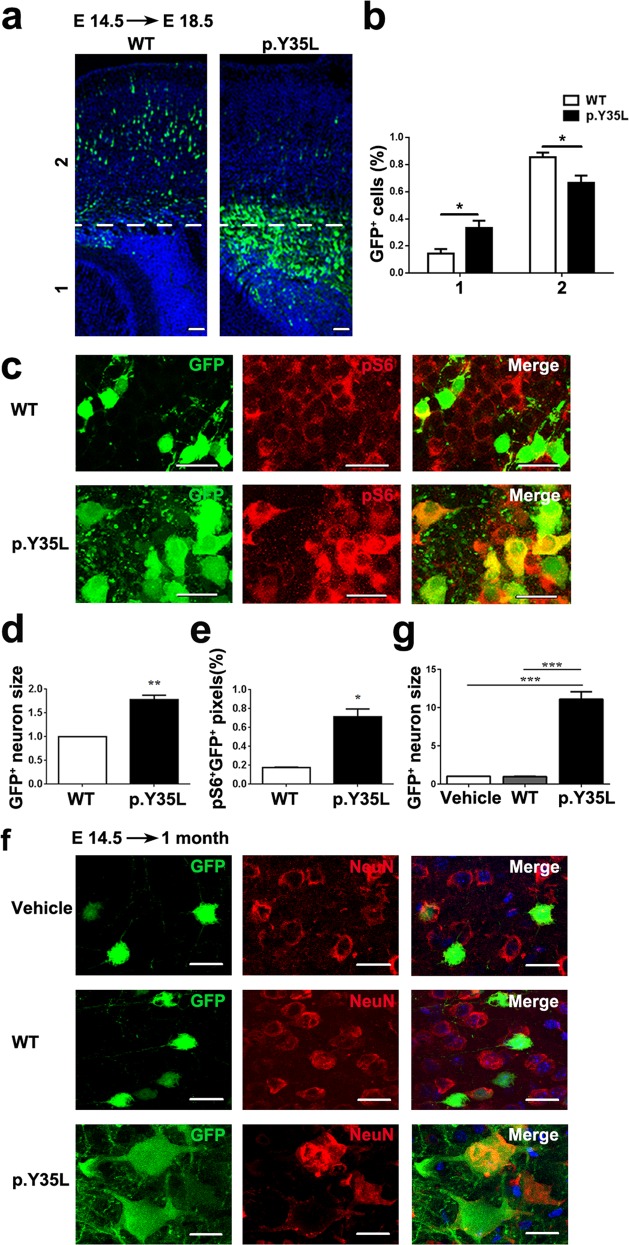
The RHEB p.Y35L mutation induces neuronal migration defects, cytomegalic neurons, and mTOR pathway activation in vivo. E14.5 mouse embryos were electroporated with control vehicle, wild-type (WT) or p.Y35L RHEB plasmids coexpressing GFP through an IRES sequence. **a** At E18.5, embryos were collected and brain coronal sections were stained with DAPI and imaged under a confocal microscope. WT and p.Y35L sample images were aligned in the same orientation and roughly defined into two bins, with bin 1 representing the regions below the cortical plate and bin 2 representing the cortical plate. GFP-positive cells are indicated in green, and nuclei are indicated in blue. Scale bars: 100 μm. **b** The percentage of GFP + neurons in each bin was quantified according to the fluorescence intensity using ImageJ. Data are presented as the mean ± s.e.m, *n* = 4, *P < 0.05 (Student’s *t*-test). **c** At E18.5, embryo brain coronal sections were immunostained using an anti-phosphorylated S6 (pS6) antibody and a corresponding secondary antibody. Images were acquired by confocal microscopy. GFP (green) and the pS6 immunoreactivities (red) were visualized as indicated. Scale bars: 20 μm. Soma sizes of GFP-positive cells (**d**) and colocalization of GFP and pS6 (**e**) as indicated by yellow convergent signals in **c** were quantified using ImageJ for comparison. Data are presented as the mean ± s.e.m, *n* = 4, *P < 0.05, **P < 0.01 (Student’s *t*-test). **f** At P30, electroporated brain samples were immunostained using an anti-NeuN antibody and a corresponding secondary antibody, and images were acquired by confocal microscopy. GFP (green) and NeuN immunoreactivity (red) were visualized as indicated. Scale bars: 20 μm. **g** Soma sizes of GFP- and NeuN-double positive cells in **f** were quantified using ImageJ. Data are presented as the mean ± s.e.m. ***P < 0.001 (Vehicle: *n* = 3, WT: *n* = 4, p.Y35L: *n* = 3, one-way ANOVA with Tukey’s post test)

We electroporated wild-type or p.Y35L RHEB IRES-GFP plasmids into E14.5 mouse embryos and measured cortical radial migration and S6 phosphorylation in GFP-positive cells at E18.5 or at approximately P30. Mice expressing p.Y35L RHEB showed decreased GFP-positive cell numbers in the cortical plate region and increased GFP-positive cell numbers in regions below the cortical plate compared with those expressing wild-type RHEB (Fig. 3a, b and Supplementary Fig. 3), suggesting that altered neuronal migration is associated with RHEB p.Y35L expression. Moreover, RHEB p.Y35L-expressing, GFP-positive neurons featured enhanced phosphorylated S6 staining (Fig. 3c, e) and an increased soma size (Fig. 3c, d, f, g), indicating that the RHEB p.Y35L mutation induces aberrant mTOR activation and cytomegalic neurons in vivo.

**Fig. 4:**
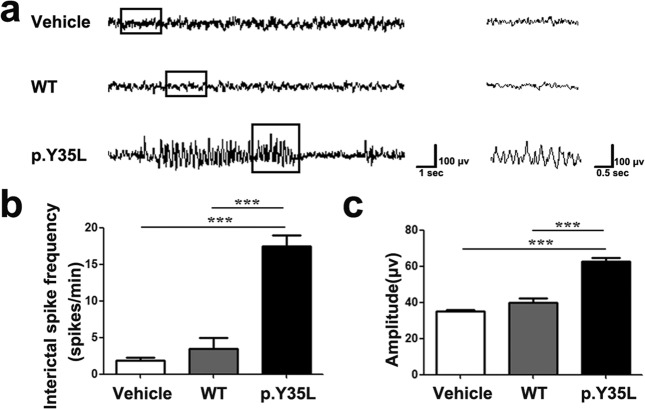
RHEB p.Y35L mutation induces abnormal EEG in mice. **a** EEG recordings of mice with embryonic expression of control vehicle, wild-type (WT) or p.Y35L RHEB plasmids. The interictal spike frequency (**b**) and amplitude (**c**) of EEG in **a** were quantified for comparison. Data are presented as the mean ± s.e.m. ***P < 0.001 (Vehicle: *n* = 3, WT: *n* = 4, p.Y35L: *n* = 3, one-way ANOVA with Tukey’s post test)

Importantly, 1-month-old mice subjected to embryonic expression of RHEB p.Y35L exhibited spontaneous tonic-clonic seizures (Supplementary Video 1), whereas mice with embryonic expression of the control vehicle vector or wild-type RHEB demonstrated no such phenomena. Moreover, mice with RHEB p.Y35L expression had an abnormal EEG (Fig. 4a) with a markedly increased interictal spike frequency (Fig. 4b) and amplitude (Fig. 4c). These results demonstrate that the p.Y35L RHEB mutation is associated with FCDII pathogenicity.

### Rapamycin treatment attenuates abnormal EEG and seizures in mice expressing the RHEB p.Y35L variant

**Fig. 5:**
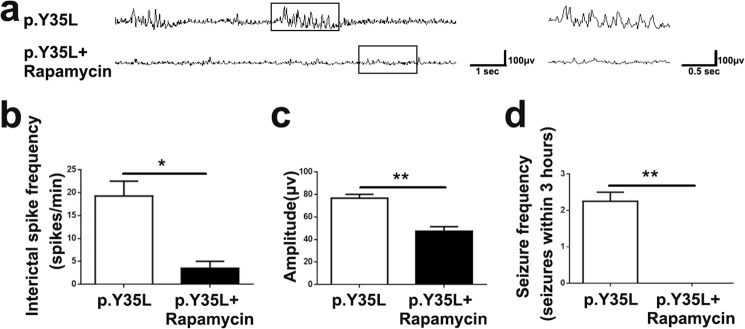
Rapamycin treatment attenuates RHEB p.Y35L mutation-induced abnormal EEG and seizures. **a** One-and-half-month-old mice with embryonic expression of the p.Y35L RHEB plasmid were first recorded for EEG, treated with rapamycin at 10 mg/kg for 11 days, and recorded for EEG again. The interictal spike frequency (**b**) and amplitude (**c**) of EEG in **a** were quantified for comparison. Data are presented as the mean ± s.e.m, *n* = 3, *P < 0.05, **P < 0.01 (Student’s *t*-test). **d** The seizure frequency in p.Y35L RHEB-carrying mice was recorded over a 3-h-long period before and after rapamycin treatment for comparison. Data are presented as the mean ± s.e.m, *n* = 3, **P < 0.01 (Student’s *t*-test)

When we treated mice expressing RHEB p.Y35L with the mTOR inhibitor rapamycin, we found that the abnormal EEG (Fig. 5a) with an increased interictal spike frequency (Fig. 5b) and amplitude (Fig. 5c) in these mice was markedly reversed. Moreover, rapamycin treatment dramatically reduced the seizure occurrence frequency in these mice (Fig. 5d). These results further confirm that the RHEB p.Y35L mutation causes FCDII by activating the mTOR pathway.

### Downregulation of MED16 reduces S6 phosphorylation

**Fig. 6:**
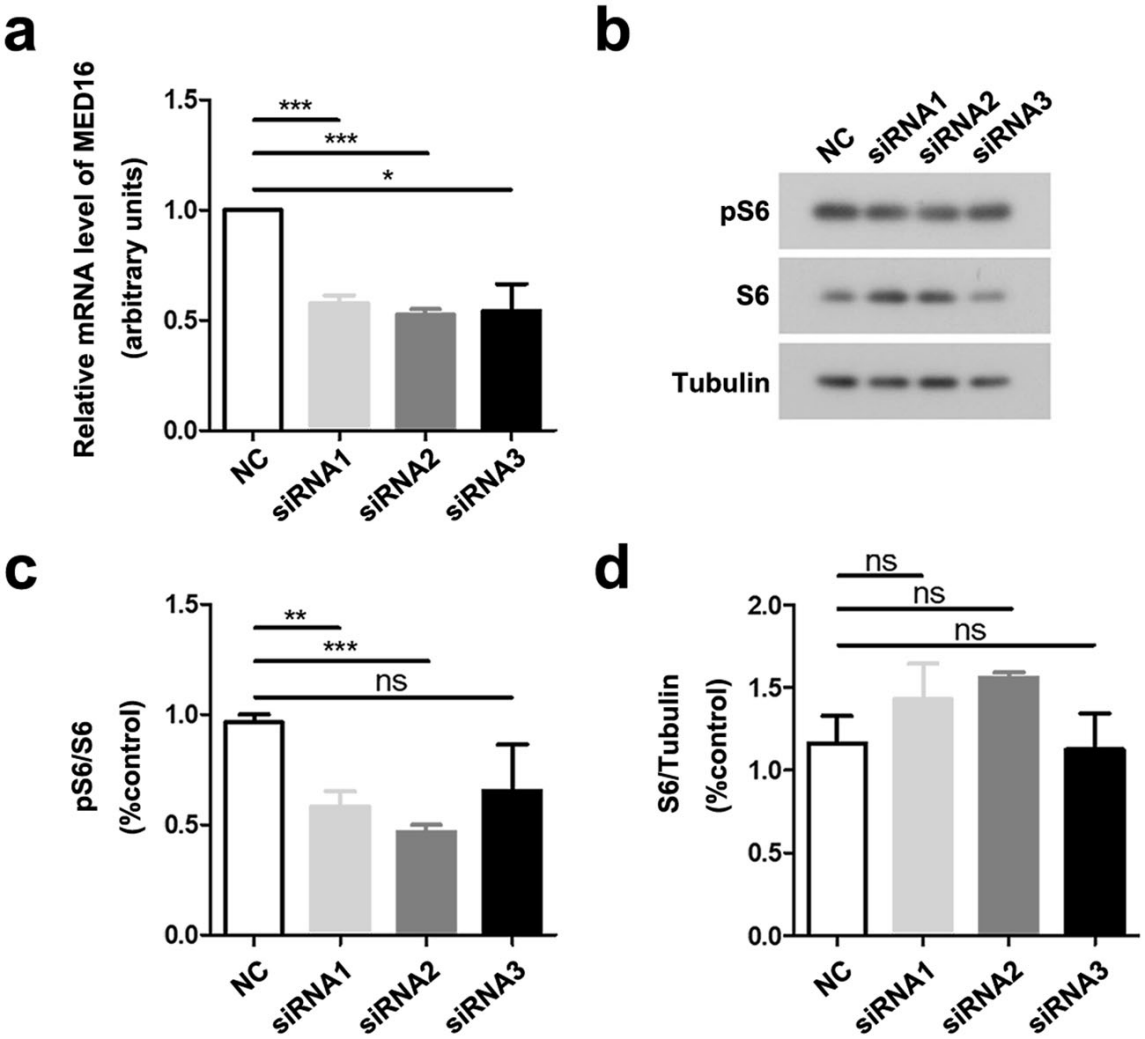
Downregulation of *MED16* reduces S6 phosphorylation. HEK293T cells were transfected with scrambled control siRNA (NC) or *MED16* siRNAs (siRNA1, siRNA2, and siRNA3) for 48 h. **a** The relative *MED16* mRNA levels in transfected cells were quantified by quantitative real-time PCR and normalized to the β-actin mRNA levels for comparison. **b** Cell lysates were analyzed by SDS-PAGE and immunoblotting to detect phosphorylated S6 (pS6), total S6 and α-tubulin. **c** The levels of pS6 were normalized to those of total S6 for comparison. **d** The levels of S6 were normalized to those of α-tubulin for comparison. Data are presented as the mean ± s.e.m, *n* = 4, *P < 0.05, **P < 0.01, ***P < 0.001, ns: not significant (Student’s *t*-test)

High-priority screening of the WES data identified one indel in *MED16* in another FCDII patient. MED16 is a conserved component within the RNA polymerase II transcriptional coactivation complex^30^. The identified *MED16* indel mutation results in a frameshift (Table 2). However, *MED16* downregulation reduced S6 phosphorylation (Fig. 6a–d). Therefore, it is unlikely that this somatic *MED16* indel mutation can trigger FCDII onset through mTOR activation.

## Discussion

RHEB functions as a crucial switch in the PI3K-AKT-mTOR pathway^6^. Here, we identified a brain somatic doublet mutation c.(A104T, C105A) in the *RHEB* gene, which resulted in the RHEB p.Y35L mutation in one FCDII patient. RHEB p.Y35 is highly conserved among divergent species. Structural analysis suggests that p.Y35 may shield the phosphate moiety of GTP in RHEB, leading to closure of the GTP-binding site^29^. Therefore, the p.Y35L mutation could potentially stabilize RHEB-GTP binding to enhance RHEB activation. Indeed, the recombinant RHEB p.Y35L mutant protein bound more GTPγS than wild-type RHEB; RHEB p.Y35L mutant expression markedly increased S6 phosphorylation, which is indicative of PI3K-AKT-mTOR pathway activation. Moreover, expression of RHEB p.Y35L in the embryonic brain during early developmental stages resulted in dysregulated neuron migration, cytomegalic neuron formation, abnormal EEG, and seizures, all of which were reminiscent of those found in patients with FCDII, suggesting that the somatic RHEB p.Y35L mutation is indeed pathogenic. One recent study found that expressing a constitutively active form of RHEB to upregulate the PI3K-AKT-mTOR pathway in mouse brains through in utero electroporation also resulted in FCDII-like hallmarks^31^, further supporting our hypothesis that brain somatic mutations in *RHEB* may be a new contributor to FCDII pathogenesis.

High-priority screening of the WES data also identified one SNV mutation in *DPP7* in the FCDII patient carrying an indel in *MED16*. As the identified *DPP7* SNV mutation was predicted to induce a change in mRNA splicing (Table 2), the resultant DPP7 product remained difficult to determine, and we did not study whether this *DPP7* somatic mutation causes FCDII.

Somatic *TSC1* mutations have been found to be associated with FCDII^10,11^. Our targeted sequencing identified a brain somatic *TSC1* missense mutation in one FCDII patient (Table 2 and Supplementary Fig. 4a, b). The high frequency of this mutation, 50.1%, in the brain might be due to it having its origin at a very early neural developmental stage. This mutation is predicted to be deleterious to the function of TSC1, implying its association with FCDII pathogenesis, which deserves further scrutiny.

In summary, we identified a brain somatic doublet mutation in the *RHEB* gene that can aberrantly activate the PI3K/AKT/mTOR pathway, thereby triggering FCDII onset. Moreover, we found that treatment with rapamycin could attenuate abnormal EEG and seizures in mice expressing this RHEB mutation, and this result is consistent with the reported rescuing effect of rapamycin on FCDII murine models expressing disease-associated mutations in *MTOR* and other related genes^13,31,32^. These findings not only further demonstrate that somatic mutations in genes within the PI3K/AKT/mTOR pathway are pathogenically associated with FCDII but also suggest that inhibition of this pathway holds potential in FCDII intervention.

## Supplementary information

Supplementary Data.

Supplementary Video 1.

## Data Availability

The raw data used to prepare the figures and tables will be made available or shared in anonymized format by request of a qualified investigator.
