# Supplementary material for: A brain somatic RHEB doublet mutation causes focal cortical dysplasia type II
Source: Exp Mol Med. 2019 Jul 23;51(7):84. doi: 10.1038/s12276-019-0277-4 (PMC6802736; doi:10.1038/s12276-019-0277-4)
Supplement: Supplementary file 1 — Supplementary Data. [file 12276_2019_277_MOESM1_ESM.doc]

**Supplementary data**

**A brain somatic *RHEB* doublet mutation causes focal cortical dysplasia type II**

Shanshan Zhao^1*^, Zhenghui Li^2,3*^, Muxian Zhang^1^, Lingliang Zhang^1^, Honghua Zheng^1^, Jinhuan Ning^1^, Yanyan Wang^1^, Fengpeng Wang^2,4^, Xiaobin Zhang^2,4^, Hexia Gan^2^, Yuanqing Wang^2^, Xian Zhang^1^, Hong Luo^1^, Guojun Bu^5^, Huaxi Xu^6^, Yi Yao^2,4,7#^ and Yun-wu Zhang^1#^

^1^ Fujian Provincial Key Laboratory of Neurodegenerative Disease and Aging Research, Institute of Neuroscience, School of Medicine, Xiamen University, Xiamen, Fujian 361102, China

^2^ Neuromedicine Center, the 174th Hospital of Chinese People’s Liberation Army, Affiliated Chenggong Hospital, Xiamen University, Xiamen, Fujian 361003, China

^3^ Department of Neurosurgery, Kaifeng Central Hospital, Kaifeng, Henan 475000, China

^4^ XiaMen Humanity Hospital, No.3777 XianYue Road, HuLi District, XiaMen, FuJian, 361015, China

^5^ Department of Neuroscience, Mayo Clinic, Jacksonville, FL 32224, USA

^6^ Neuroscience Initiative, Sanford-Burnham-Prebys Medical Discovery Institute, La Jolla, CA 92037, USA

^7^ Department of Pediatric Neurology, Shenzhen Children's Hospital, Shenzhen 518026, Guangdong Province, China.

^*^These authors contributed equally to this work.

^#^Correspondence to: Yun-wu Zhang (E-mail: yunzhang@xmu.edu.cn; Tel: 86-592-2188528), or Yi Yao (13656008777@163.com; Tel: 86-592-5262115).

**Supplementary Materials and Methods**

**RHEB siRNA transfection**

HEK293T cells were first transfected with the RHEB Y35L mutant plasmid. After equally splitting, cells were transfected with a scrambled control siRNA (sense 5’-UUCUCCGAACGUGUCACGUTT-3’) or a siRNA (sense 5’-CUCCUUAGAUCCAACCAUATT-3’) targeting the mutant RHEB for 48h. Cell lysates were analyzed by western blotting.


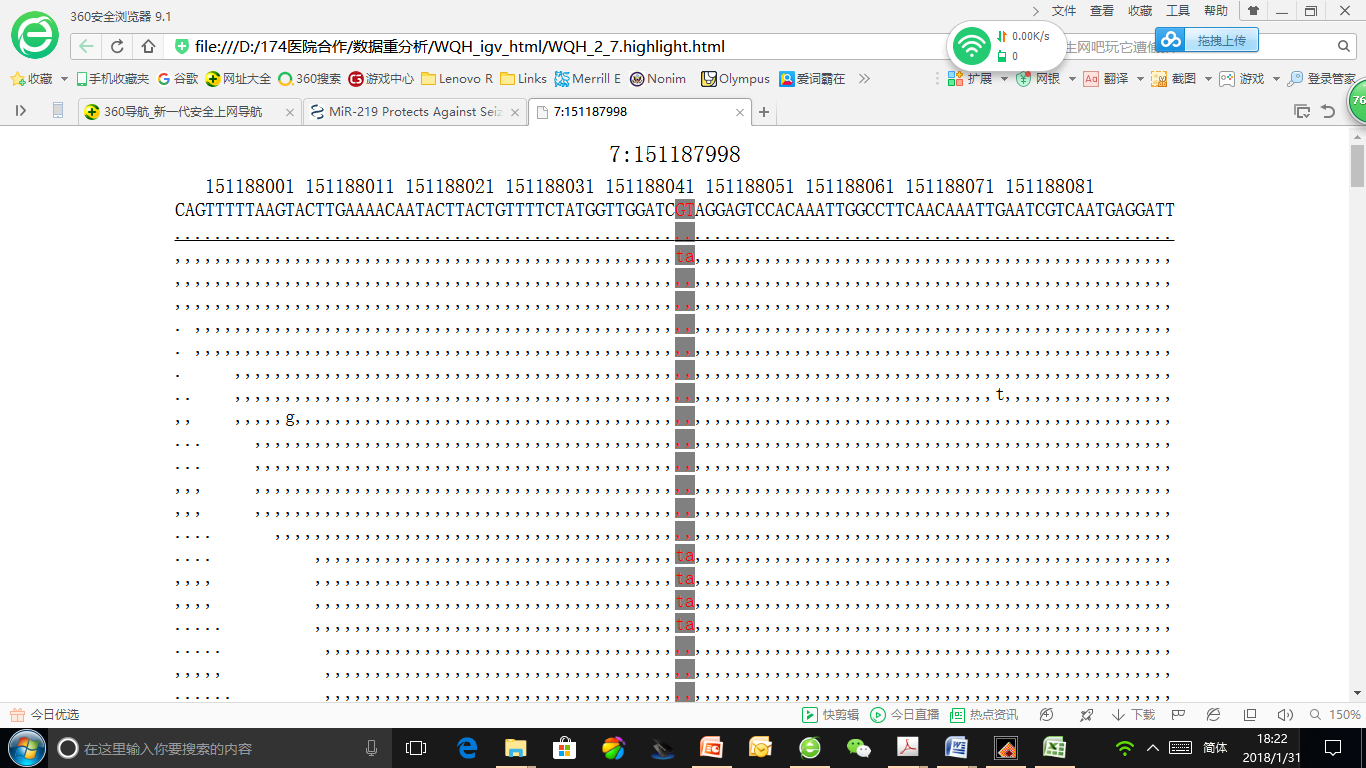


**Supplementary Fig. 1 IGV analysis of *RHEB* mutation sites from WES reads of the FCDII-1 brain sample (only partial reads were shown)**

The results show that G151188048T and T151188049A mutations in the *RHEB* gene occur concurrently.


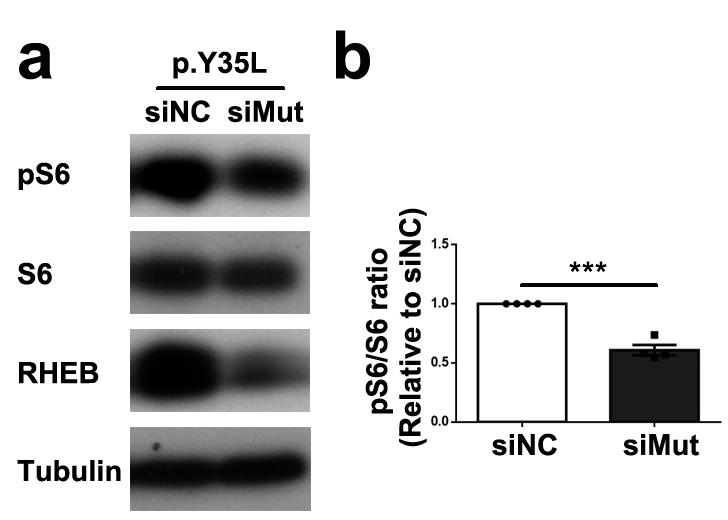


**Supplementary Fig. 2 Downregulating RHEB p.Y35L expression reduces S6 phosphorylation**

**a** HEK293T cells were first transfected with the RHEB Y35L mutant plasmid, and then transfected with a scrambled control siRNA (siNC) or a siRNA targeting the mutant RHEB (siMut). Equivalent protein quantities from the cell lysates were subjected to immunoblotting to detect phosphorylated S6 (pS6), total S6, RHEB and α-tubulin. **b** Protein levels of pS6 were quantified by densitometry and normalized to those of total S6 for comparison, where pS6/total S6 ratios of siNC samples were set as one arbitrary units. Data represent the mean±s.e.m, n=4, ^***^*P*< 0.001 (Students’ t-test).


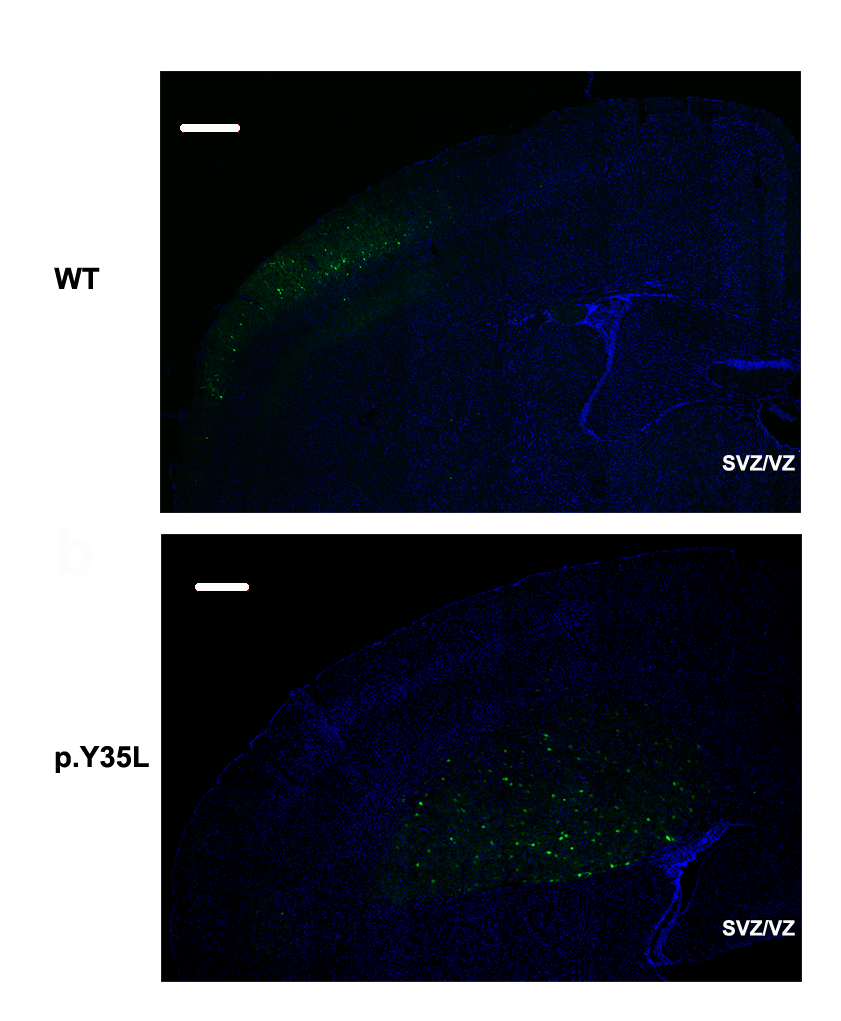


**Supplementary Fig. 3 RHEB p.Y35L mutation induces neuronal migration defects**

E14.5 mouse embryos were electroporated with wild type (WT) or p.Y35L RHEB plasmids co-expressing GFP through an IRES sequence. At P30, mouse brains were collected and brain sections were stained with DAPI and imaged under a confocal microscope. Scale bars: 500 μm.


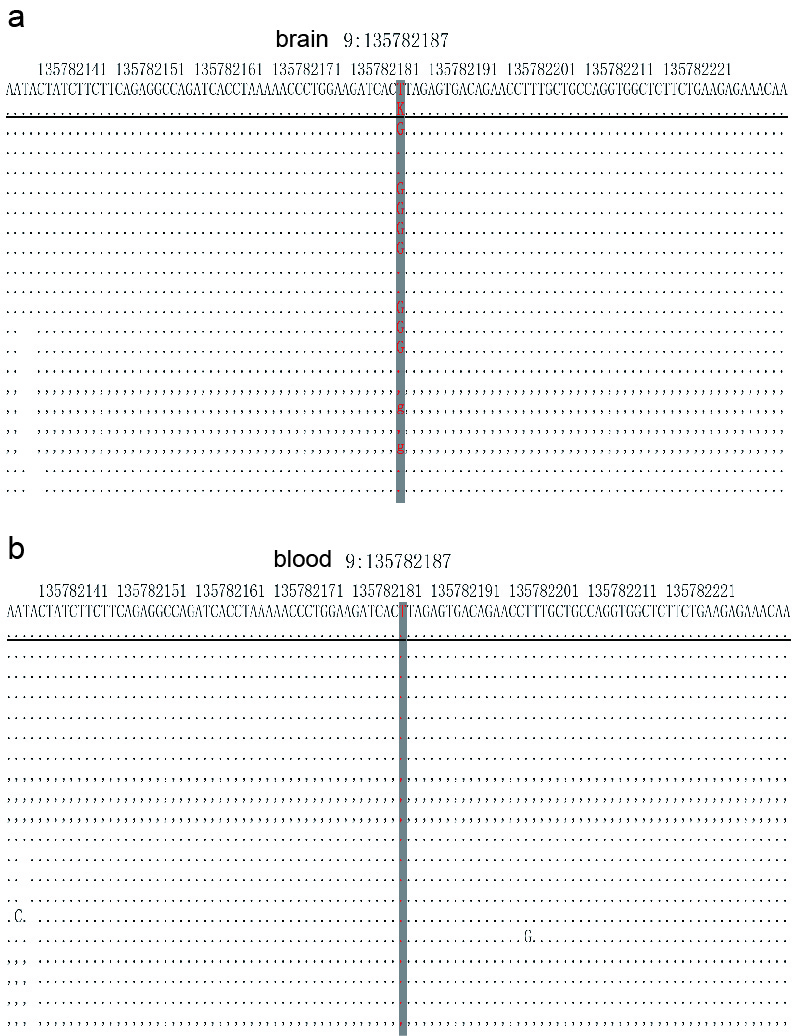


**Supplementary Fig. 4 IGV analysis of the *TSC1* mutation site from WES reads of FCDII-5 brain and blood samples (only partial reads were shown)**

**a, b** The results show that T135782187G mutation in the *TSC1* gene occurs at a frequency of 50.1% in the brain (**a**) but nearly zero in the blood (**b**).

**Supplementary Video 1 Mice subjected to embryonic expression of the RHEB p.Y35L variant exhibit spontaneous tonic-clonic seizures**

ICR mice at E14.5 were subjected to *in utero* electroporation. At P30, they were recorded for seizure behaviors. The mouse who stayed at the right lower corner at the beginning was subjected to embryonic expression of the RHEB p.Y35L variant and developed spontaneous tonic-clonic seizures. The other mouse was a littermate control.

**Supplementary Table 1 Summary of deep WES data and identified mutation numbers in protein-encoding genes^a^**

| **Sample** | **Raw data size (G)** | **Average sequencing depth on target (X)** | **SNV missense** | **SNV stopgain** | **SNV stoploss** | **SNV splicing** | **Indel frameshift deletion** | **Indel frameshift insertion** | **Indel nonframeshift deletion** | **Indel nonframeshift insertion** | **Indel stopgain** | **Indel stoploss** | **Indel splicing** |
| --- | --- | --- | --- | --- | --- | --- | --- | --- | --- | --- | --- | --- | --- |
| FCDII-1_  blood | 63.13 | 760.04 | 9111 | 72 | 8 | 483 | 14 | 11 | 54 | 51 | 0 | 0 | 48 |
| FCDII-1_  brain | 50.47 | 624.85 | 9095 | 72 | 8 | 485 | 14 | 12 | 50 | 44 | 0 | 0 | 58 |
| FCDII-2_  blood | 53.27 | 638.60 | 9147 | 61 | 11 | 491 | 17 | 17 | 80 | 81 | 2 | 1 | 61 |
| FCDII-2_  brain | 63.69 | 802.85 | 9125 | 63 | 10 | 494 | 12 | 10 | 51 | 55 | 0 | 1 | 41 |
| FCDII-3_  blood | 62.30 | 754.25 | 9100 | 66 | 7 | 500 | 13 | 10 | 45 | 49 | 0 | 1 | 44 |
| FCDII-3_  brain | 57.18 | 726.86 | 9072 | 68 | 7 | 501 | 10 | 11 | 45 | 44 | 1 | 1 | 50 |
| FCDII-4_  blood | 50.31 | 603.56 | 9152 | 61 | 9 | 480 | 19 | 15 | 65 | 63 | 3 | 2 | 62 |
| FCDII-4_  brain | 57.24 | 691.62 | 9168 | 59 | 8 | 486 | 18 | 13 | 52 | 52 | 2 | 2 | 56 |

^a^Only mutation numbers of SNVs and indels in protein-encoding genes that would alter protein sequences were listed.

**Supplementary Table 2 Brain somatic mutations identified by WES**

| **Sample**  (brain vs. blood) | **SNV missense** | **SNV stop gain** | **SNV stop loss** | **SNV splicing** | **Total SNV numbers**^a^ | **Indel frameshift deletion** | **Indel frameshift insertion** | **Indel non-**  **frameshift deletion** | **Indel non-**  **frameshift insertion** | **Indel stop gain** | **Indel stop loss** | **Indel splicing** | **Total Indel numbers**^a^ | **CNV gain count** | **CNV gain size** | **CNV loss count** | **CNV loss size** | **Total CNV numbers**^a^ |
| --- | --- | --- | --- | --- | --- | --- | --- | --- | --- | --- | --- | --- | --- | --- | --- | --- | --- | --- |
| FCDII-1 | 1 | 1 | 0 | 0 | 371 | 0 | 0 | 0 | 0 | 0 | 0 | 0 | 12 | 66 | 1389883 | 0 | 0 | 66 |
| FCDII-2 | 2 | 0 | 0 | 1 | 328 | 1 | 0 | 0 | 0 | 0 | 0 | 0 | 5 | 113 | 25149180 | 2 | 96252 | 115 |
| FCDII-3 | 0 | 0 | 0 | 0 | 286 | 0 | 0 | 0 | 0 | 0 | 0 | 0 | 2 | 13 | 541263 | 3 | 230253 | 16 |
| FCDII-4 | 3 | 0 | 0 | 0 | 276 | 0 | 0 | 0 | 0 | 0 | 0 | 0 | 10 | 31 | 18031 | 7 | 4257 | 38 |

^a^Total numbers include somatic mutations in non-CDS regions and in non-protein-encoding genes.
